# Experimental and Localisation Method for Non-Destructive Testing of Cable Corrosion Based on Weak Magnetic Imaging

**DOI:** 10.3390/s26041250

**Published:** 2026-02-14

**Authors:** Yujie Wu, Runchuan Xia, Yuanzheng Feng, Youjia Yang, Houxuan Li, Mingyang Li

**Affiliations:** 1 State Key Laboratory of Mountain Bridge and Tunnel Engineering, Chongqing Jiaotong University, Chongqing 400074, China; wuyujie@mails.cqjtu.edu.cn (Y.W.); yzfeng@mails.cqjtu.edu.cn (Y.F.); 622240080022@mails.cqjtu.edu.cn (Y.Y.); hxli@mails.cqjtu.edu.cn (H.L.); weich88541@163.com (M.L.); 2 School of Civil Engineering, Chongqing Jiaotong University, Chongqing 400074, China

**Keywords:** parallel steel wire rope, spontaneous magnetic leakage, corrosion, weak magnetic imaging, threshold

## Abstract

In order to address the challenge of accurately identifying the extent of corrosion in parallel steel wire cables, a series of corrosion detection tests were performed on parallel wire bundles with varying degrees of corrosion. Following the collection of weak magnetic signals from a 12-channel Hall array, the influence patterns of corrosion severity on the distributions (*B_x_*, *B_z_*) of leakage magnetic signals were analysed. The experimental results were validated by means of a three-dimensional finite element model, leading to the proposal of a novel weak magnetic imaging method based on the fusion of adaptive threshold *K* and linear interpolation. The findings of the study demonstrate a strong linear relationship (R^2^ = 0.998) between axial corrosion length and the peak-to-trough distance of the normal component *B_z_*. Furthermore, it was determined that a positive correlation exists between the circumferential corrosion width and the circumferential distribution range of *B_z_*. The utilisation of an adaptive threshold *K* for the purpose of threshold segmentation has been demonstrated to be an effective method for the delineation of corrosion boundaries, thereby enabling precise localisation. The present research provides technical support for the visualisation and quantitative assessment of cable corrosion.

## 1. Introduction

It is evident that as economic construction has undergone progressive development, there has been an annual increase in the volume of transport infrastructure. Cable-stayed bridges, a highly representative bridge structure type, have gained widespread application globally due to their structural efficiency, aesthetic appeal, and economic viability [1]. In this context, the stay cables—the core load-bearing components that transmit bridge weight and traffic loads—are perpetually exposed to complex environmental conditions. Prolonged exposure to various environmental factors has been demonstrated to compromise the integrity of bridge components, rendering them susceptible to corrosion [2], fatigue, and other forms of damage [3,4]. This poses a significant threat to the structural integrity and safety of bridges [5]. A particular challenge is the detection of corrosion within the parallel steel wires, as the protective sheathing obscures the internal corrosion damage. Consequently, the issue of corrosion detection in stay cables remains a persistent challenge within the industry [6].

In order to address the challenge of detecting corrosion in suspension cables, researchers have explored various non-destructive testing methods with a view to diagnosing internal damage within bridge cables. Non-destructive testing techniques are known to be problematic due to a number of factors. Firstly, it is important to note that conventional techniques, including acoustic emission testing [7,8], radiographic testing [9,10], infrared thermography [11,12] and ultrasonic testing [13,14], are frequently inadequate in terms of efficiency. Secondly, these techniques are expensive and lack sensitivity to internal damage. Furthermore, the efficacy of these measures is considerably influenced by the practical constraints inherent to on-site inspection environments.

Doubov [15] built upon the theoretical foundations of leakage flux detection technology and integrated ferromagnetic physics. This development led to the proposal of Metal Magnetic Memory (MMM) technology, which was introduced into the field of non-destructive testing research. Self-magnetic Flux Leakage (SMFL) [16] is a pioneering method that functions independently of external magnetic fields. The device utilises the Earth’s magnetic field to analyse stress concentrations, defect locations and damage severity by measuring flux leakage variations across ferromagnetic material surfaces [17]. This approach has been demonstrated to reduce inspection costs in practical engineering applications. Due to its high sensitivity to early-stage damage in ferromagnetic materials, SMFL technology has been extensively applied in the following areas: oil and gas pipeline inspection [18]; non-destructive assessment of railway tracks [19]; tank bottom plate inspection [20]. In recent years, scholars have conducted extensive research on the damage conditions of primary ferromagnetic components (reinforcing bars, steel wires, and steel strands) in various bridge engineering projects. Pang [21] and Tong et al. [22,23,24] proposed an SMFL-based assessment method for the load-carrying capacity of reinforced concrete bridges by analysing the correlation between changes in the mechanical properties of reinforcing bars and SMFL signals. This objective was realised through the analysis of the correlation between alterations in the mechanical properties of reinforcing bars and SMFL signals. The contribution of Zhou [24] and Zhang [25,26] to the field entailed the proposition of a dual-defect magnetic dipole model. The theoretical framework has been developed to address the issue of cable strand breakage. The present model examined the relationship between magnetic signal characteristics and influencing factors. Xia [27,28,29] conducted a series of stress-free corrosion detection tests on steel strands. The MMM approach was employed as the methodological framework. This approach combined the SMFL theoretical equation for corrosion zones. The proposal further encompasses the utilisation of spatial localisation methodologies and quantitative characterisation analyses for the identification of corroded regions. In view of the weak magnetic detection environment that is characteristic of real parallel-wire-stranded long-span bridges, Hall sensors were selected for the purpose of acquiring magnetic signal data in this experiment. The Hall sensor array has been demonstrated to be capable of stable operation for extended periods when powered by batteries, even in conditions of high altitude, salt fog and impact. The elimination of the velocity dependency inherent in coil-type sensors ensures high performance and engineering feasibility across four critical dimensions: static operation, weak magnetic response, array deployment, and field implementation [30,31]. This facilitates the efficient detection of wire damage [32].

The employment of sophisticated signal processing algorithms has facilitated the evolution of non-destructive testing methodologies, thus enabling the acquisition of structural damage images. In the domain of imaging performance optimisation, Anoni et al. [33] employed ICC metrics and IDW interpolation to enhance ultrasonic testing methodologies. In addressing the efficiency challenges of data acquisition in infrared thermal imaging, Liu et al. [34] proposed a slow feature thermal imaging method based on self-guided filtering. Sun et al. [35] further enhanced the signal-to-background ratio (SBR) and defect signal amplitude of magnetic flux leakage (MFL) detection through the optimisation of sensor structural parameters, generating preliminary images of broken wires within wire bundles. However, while existing spontaneous Self-magnetic Flux Leakage (SMFL) techniques have been shown to localise corrosion damage through multiple feature parameters and predict corrosion width ranges [36], they are unable to accurately quantify corrosion morphology and damage severity due to mutual interference between magnetic signals from multiple wires within parallel steel cables [37]. Consequently, there is an urgent need for a weak magnetic imaging approach to visually characterise corrosion zones within parallel steel cables.

Parallel steel wire cables were investigated in this study through the employment of electrochemical corrosion tests to simulate various damage conditions. The integration of magnetic signal acquisition experiments with multi-physics coupled simulations enables a systematic exploration of the mapping relationship between corrosion parameters and weak magnetic signals. The present paper proposes a novel approach to magnetic imaging, founded upon gradient standard deviation. The employment of PyCharm software facilitates the precise localisation of corrosion zones and the quantification of damage severity. This provides theoretical foundations and technical support for the maintenance and management of bridges.

## 2. Experimental Scheme

### 2.1. Test Materials

The steel wire bundle utilised in the test comprised 37 strands of 7 mm-1670 high-strength steel wire. The specimen measured 80 centimetres in length and 7 millimetres in diameter. The chemical composition and mechanical properties of the material are outlined in Table 1.

Two experimental conditions—axial corrosion length (Group A) and circumferential corrosion width (Group B)—were established through the use of electrochemical accelerated corrosion testing. The objective of this study was to systematically investigate the weak magnetic array signal characteristics of parallel steel wire bundles under varying corrosion states. A methodology for identifying axial and circumferential corrosion areas was proposed on the basis of correlation analysis of weak magnetic signal parameters within the corrosion zones. This method provides critical data support for subsequent weak magnetic imaging techniques. The precise test parameters are delineated in Table 2.

### 2.2. Test Equipment and Procedure

(1) Test setup

**Figure 1:**
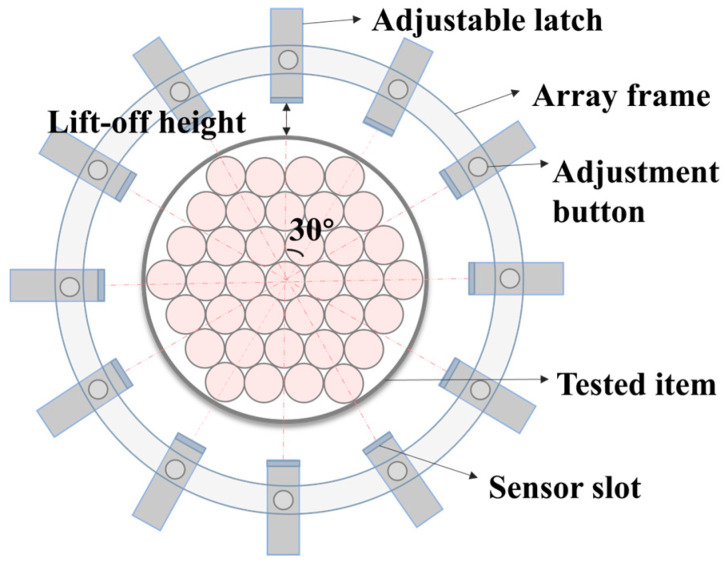

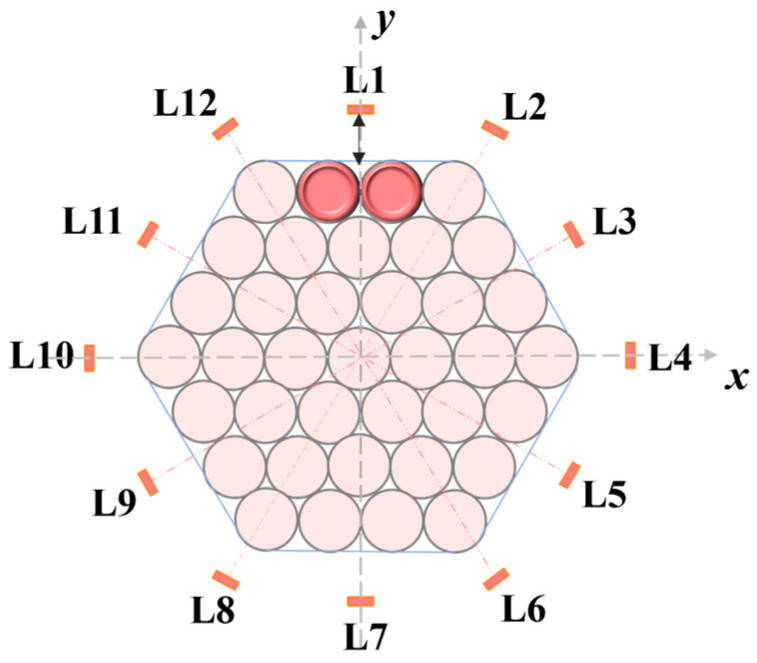
Schematic diagram of the sensor array device.

The sensor mounting apparatus employed in this study was a ring-shaped array device with a diameter of 60 mm. Twelve Honeywell HMC5883L magnetometers were distributed uniformly at 30° equiangular intervals, thus forming twelve standardised scanning paths designated L1–L12, as shown in Figure 1. The apparatus under discussion achieves precise detection of magnetic field strength and direction through the anisotropic magnetoresistance effect. The multi-path synchronous acquisition design ensures spatial continuity in axial and circumferential magnetic field gradient measurements, providing reliable hardware support for subsequent weak magnetic signal analysis.

(2) Test procedure

**Figure 2:**
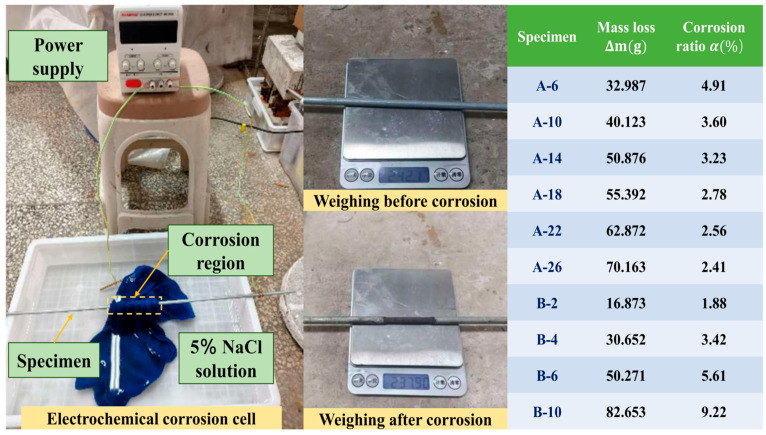
Electrochemical corrosion device.

Thirty-seven steel wires, each exhibiting identical magnetic properties, were consolidated into a single wire bundle, with a total of ten such bundles being prepared. Each bundle was assigned a unique identifier and the initial weight of the wires to be corroded within each bundle was meticulously documented. The present study employed an electrochemical accelerated corrosion method (Figure 2), the process of which entailed subjecting the 37 wire bundle specimens to controlled corrosion in a 5% NaCl solution via a 0.5 A constant current source across six stages. Subsequent to the completion of the corrosion process, the wires that had undergone corrosion were retrieved, thoroughly washed, allowed to dry in the ambient atmosphere, and weighed. The localised corrosion rate *α* was calculated for each specimen using the corrosion rate Formula (1).

(1) $$\alpha =\frac{(m_{0}-m_{1})\cdot l_{0}}{n\cdot m_{0}\cdot l}=\frac{\Delta m\cdot l_{0}}{n\cdot m_{0}\cdot l}$$

In the equation, m_0_ denotes the mass of the steel wire prior to corrosion; m_1_ denotes the mass of the steel wire after corrosion; Δm denotes the mass loss of the steel wire between corrosion and post-corrosion; *l*_0_ denotes the total length of the steel wire; *l* denotes the length corroded; n denotes the number of steel wires within the specimen.

**Figure 3:**
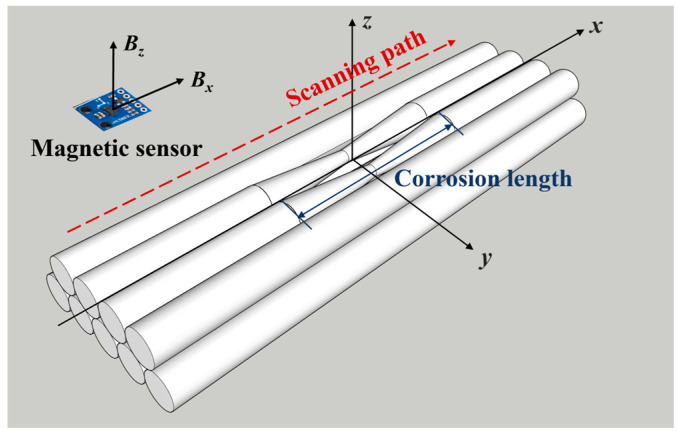
Schematic diagram of the magnetic signal acquisition path.

**Figure 4:**
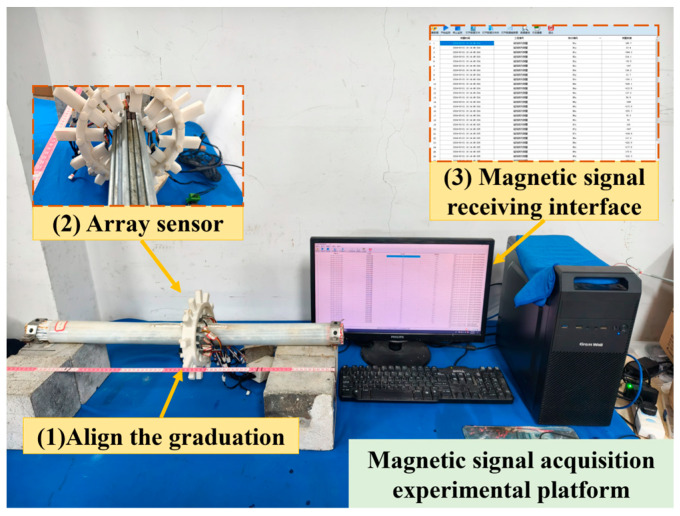
Magnetic signal acquisition test platform.

A one-dimensional scan was conducted along the length of the steel wire bundle, utilising an array sensor. The scan was conducted at a constant step size of 5 mm. The two-dimensional magnetic flux density vector ***B*** = (*B_x_*,*B_z_*) was simultaneously acquired and transmitted at each point on the surface of the bundle, where *x* and *z* correspond to the tangential and normal directions of the bundle, respectively. The scan results are defined as positive in the direction aligned with the coordinate axes (Figure 3). The utilisation of the magnetic signal acquisition test platform (Figure 4) facilitates the acquisition of the initial magnetic signal and the post-corrosion magnetic signal values of the steel wire bundle. The derivation of weak magnetic changes is achieved by calculating the difference in magnetic signals before and after corrosion.

## 3. Experimental Data Analysis

### 3.1. Axial Magnetic Signal Analysis

**Figure 5:**
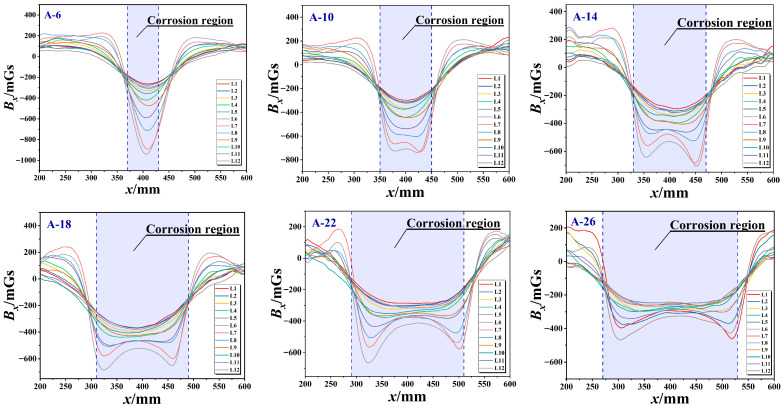
*B_x_* axial distribution diagram of different corrosion lengths.

**Figure 6:**
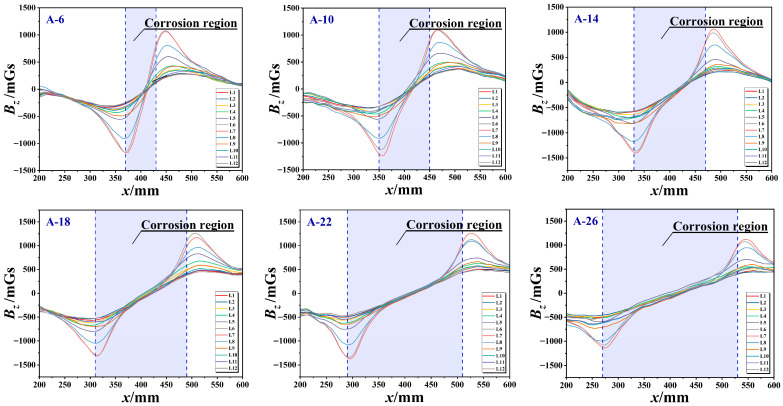
*B_z_* axial distribution map with different corrosion lengths.

By scanning the specimens in Group A sequentially from left to right, the variation patterns of magnetic signals along the tangential component *B_x_* and normal component *Bz* were obtained across 12 paths (Figure 5 and Figure 6). In this context, the horizontal coordinate *x* is indicative of the scanning region along the axial direction of the steel wire bundle, while the vertical coordinates represent the spontaneous leakage magnetic flux density Bx and the normal magnetic flux density *Bz*, respectively.

A thorough analysis of specimen *B_x_* from Group A reveals that, in instances where the corrosion length *l* ≥ 10 cm, the Bx signal across all specimens manifests a symmetrical double-trough pattern within the corrosion zone. The midpoint between the two troughs approaches the centre of the corrosion area. As the corrosion length of the wire bundle increases, the distance between the two troughs concomitantly increases, thus demonstrating a correlation between trough spacing and corrosion length. Although specimen A-6 exhibited a single-trough pattern, its trough amplitude (−832 mGs) was significantly lower than that of the other specimens. The analysis indicates that this is attributable to inadequate corrosion length, resulting in visual overlap between the two troughs.

The analysis of the *B_z_* values from Group A specimens reveals that the normal magnetic induction intensity *Bz* for all groups displays a single peak-single trough characteristic. The centres of the peak and trough coincide with the geometric centre of the corrosion zone (*x* = 400 mm). As the corrosion length increases, the distance between the peak and trough widens, with the transition becoming progressively smoother. Concurrently, signal fluctuations in the non-corrosion region remain within ±100 mGs, indicating excellent stability of the detection results. It can thus be concluded that the axial corrosion width demonstrates a positive correlation with both the horizontal distance between the midpoints of the peak and trough coordinates for the tangential component *B_x_*, and the lateral distance between the peak and trough of the normal component *B_z_*.

**Figure 7:**
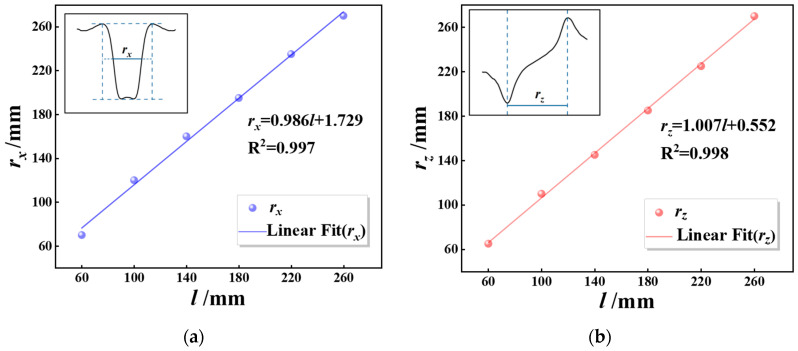
Quantitative relationship between parameter spacing and corrosion length. (**a**) Midpoint spacing of peak-to-peak values r_x_. (**b**) Peak-to-valley distance r_z_.

The findings of the research indicate a significant linear relationship between the corrosion length *l* and the characteristic parameters of the magnetic signal (Figure 7). The fitted relationship between the distance *r_x_* between the midpoints of the two peak-to-trough values and the corrosion length *l* is given by *r_x_* = 0.98*l* − 0.21 (R^2^ = 0.997). The relationship between the peak-to-peak distance *r_x_* and the corrosion length *l* is given by the following equation: *r_z_* = 1.02*l* + 0.15 (R^2^ = 0.998). A comparative analysis indicates that the *r_z_* parameter not only exhibits superior goodness-of-fit (R^2^ = 0.998) but also possesses a slope closer to the ideal value of 1. This demonstrates that the normal component *B_z_* is more suitable than the tangential component *B_x_* for quantitatively characterising axial corrosion zones. Consequently, it is recommended that the normal component *B_z_* be prioritised for corrosion zone localisation in subsequent imaging analyses.

### 3.2. Circumferential Magnetic Signal Analysis

**Figure 8:**
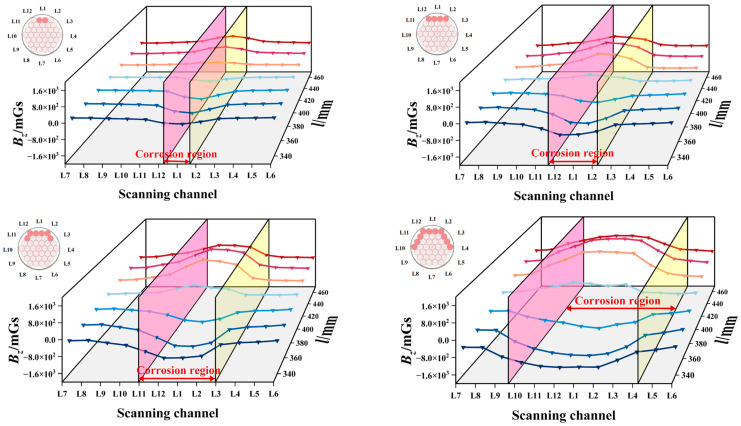
Circumferential distribution of *B_z_* for different corrosion widths.

A detailed examination of the magnetic signal results, as shown in Figure 8, was conducted by subtracting the initial magnetic field from the normal component of Group B specimens. It is evident that the absolute value of the magnetic signal at peak-to-peak intervals increases significantly with the circumferential corrosion width, and the signal change gradient exhibits a non-linear positive correlation with corrosion width. The signals across all 12 detection channels demonstrate consistent variation within the corrosion zone, with peak-to-peak spacing errors relative to the actual corrosion length measuring less than 5%. In order to investigate the relationship between corrosion width and magnetic signals, data from the 12 channels at peak-to-peak intervals were selected for analysis.

**Figure 9:**
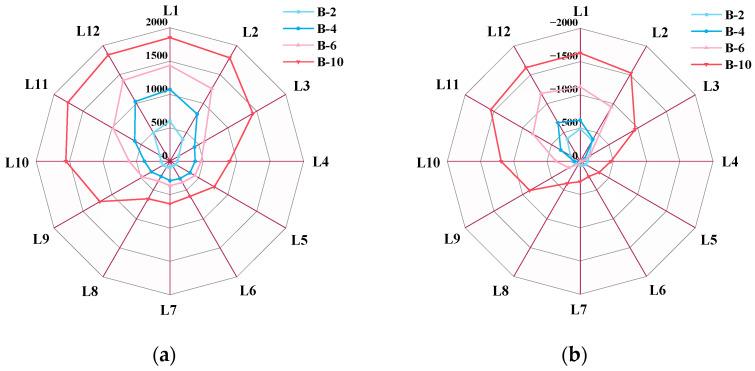
Circumferential distribution of the normal component *B_z_* at peak and trough locations. (**a**) Circumferential magnetic signal at the peak. (**b**) Circumferential magnetic signal at the trough.

The research findings indicate that the normal component *B_z_* of the 12-channel magnetic signal at both wave peaks and troughs in Group B specimens exhibits an outward expansion characteristic as corrosion width increases, as shown in Figure 9. The circumferential expansion direction is directly proportional to the circumferential corrosion width. A thorough investigation into the relationship between this circumferential expansion direction and circumferential corrosion width is of significant importance for the advancement of weak magnetic imaging technology, representing a key research direction within this field.

## 4. Simulation

### 4.1. Establishment of Finite Element Models

**Figure 10:**
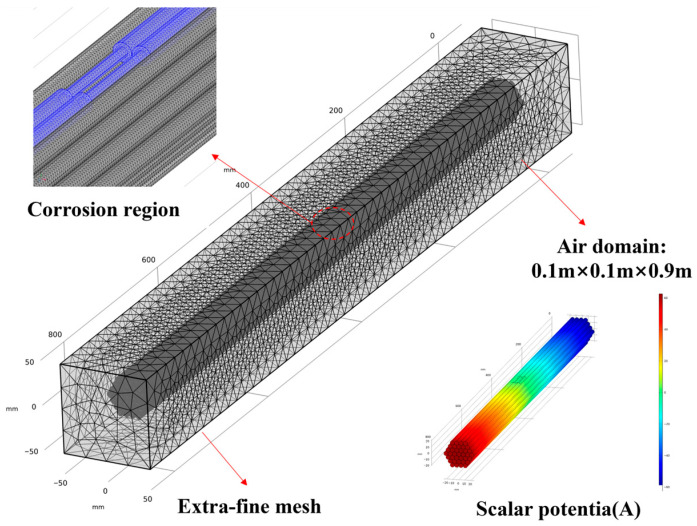
Geometric configuration of the model.

In order to validate the reliability of experimental results and further analyse the relationship between circumferential weak magnetic signals and corrosion severity, a steady-state study was conducted using the ‘Magnetic Field, No Current’ physics domain within finite element simulation software. The model was constructed based on experimental design parameters, employing 37 cylindrical specimens of 7 mm diameter and 80 cm length as scaled-down models. The simulation of uniformly corroded parallel wire bundles was achieved through the subtraction step in Boolean operations, thereby replicating the corrosion damage defects observed in the experimental parallel wire bundles. In order to replicate the experimental environment, an external air domain was defined as a rectangular volume measuring 0.1 m × 0.1 m × 0.9 m, with the parallel wire bundle model positioned at its centre. In accordance with the principle of magnetic flux conservation, the magnetisation intensity was set to 1000 A/m. The mesh size was set to “ultra-fine” and a mesh convergence study was conducted to ensure that the variation in results was less than 1%. The model geometry is shown in Figure 10.

### 4.2. Simulation Data Analysis

**Figure 11:**
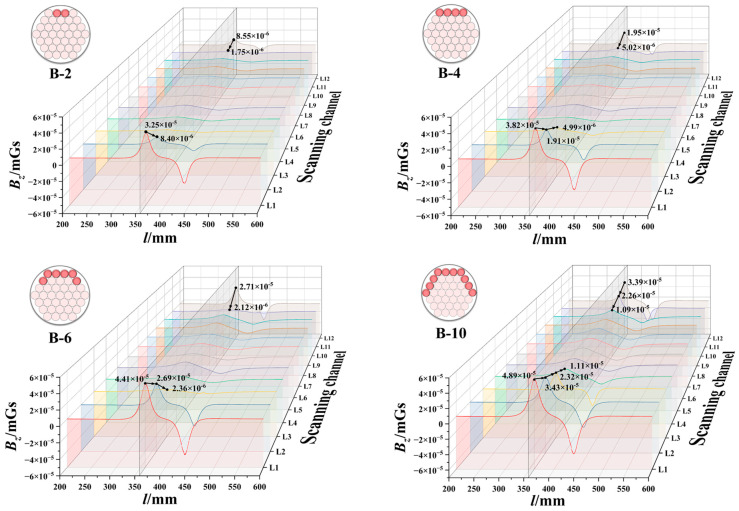
Normal component *B_z_* under different circumferential corrosion widths.

Through the utilisation of simulation studies that modelled actual operating parameters for Group B, a discernible quantitative relationship was identified between the characteristics of the normal component *Bz* signal and the circumferential corrosion width, as illustrated in Figure 11. The simulation data indicates that as the circumferential corrosion width increases from B-2 to B-10, fluctuations in the normal magnetic signal exhibit significant amplification at the corrosion centre (L1 channel). This displays a characteristic pattern of diffusion from the centre towards both sides. Concurrently, a sharp drop in magnetic signal peaks is observable at the corrosion zone boundary, consistent with experimental findings. This feature provides a reliable indicator for the precise identification of corrosion boundaries.

**Figure 12:**
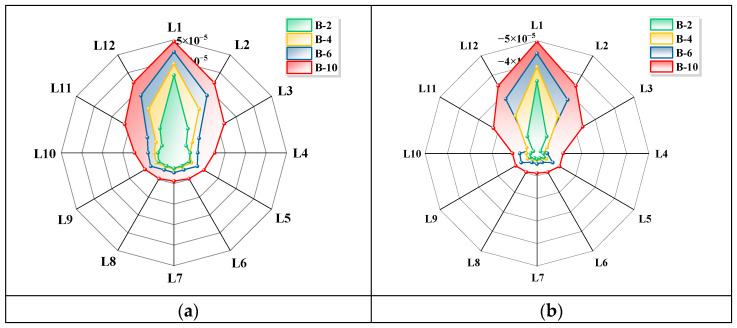
Simulation results of the normal component *B_z_* for different circumferential corrosion widths. (**a**) Circumferential magnetic signal at the peak. (**b**) Circumferential magnetic signal at the trough.

A comprehensive analysis of the circumferential signals at the peak and trough values of the simulated magnetic induction intensity *Bz* (Figure 12) reveals a pronounced outward expansion trend of the magnetic signal strength from the corrosion centre (near L1). Furthermore, the expansion amplitude exhibited a positive correlation with the corrosion width. The most pronounced expansion is observed at the predefined corrosion centre, diminishing symmetrically along adjacent azimuths (L2, L12) to form a characteristic ‘fan-shaped’ expansion pattern.

## 5. Imaging Method and Localisation Research

### 5.1. Threshold Selection

In this study, an adaptive threshold determination method based on the spatial variability characteristics of magnetic signal gradients is presented. A statistical analysis of the magnetic signal gradient fields between corroded and uncorroded regions has revealed significant spatial variability in the magnetic signal gradient ∇*B_z_* in corroded areas, whilst the gradient field in uncorroded regions remains relatively stable. The experimental data and simulation results obtained from the study provide unequivocal evidence of a precipitous decline in magnetic signal peaks at the boundaries of corroded regions. This phenomenon gives rise to markedly discrete statistical characteristics within the magnetic signal data of corroded areas. The quantification of this feature is achieved by means of standard deviation, which functions as an indicator of data dispersion. A larger standard deviation indicates a distribution of data points that is more scattered. Conversely, a smaller standard deviation indicates data points that are more concentrated around the mean. It is therefore proposed that a method for selecting threshold *K* be employed. Firstly, the standard deviation, denoted by *σ*_∇_*_Bz_*, of the gradient value, represented by ∇*B_z_*, must be calculated within the corrosion length range for each individual channel. The mean value *μ*′ of *σ*_∇_*_Bz_* across all channels is selected as the preliminary threshold, and the standard deviation *σ*′ of all 12 *σ*_∇_*_Bz_* values is added to form the threshold variable. The threshold *K* is utilised as an indicator for the classification of the severity of spontaneous leakage magnetic field variations, calculated as follows:

(2) $$\nabla B_{z}=\left(\frac{\partial B_{z}}{\partial x_{1}},\frac{\partial B_{z}}{\partial x_{2}},\ldots ,\frac{\partial B_{z}}{\partial x_{n}}\right)$$

(3) $$\sigma_{\nabla B_{z}}=\sqrt{\frac{1}{n}\sum_{i=1}^{n}\left(\nabla B_{zi}-\mu \right)}$$

(4) $$\mu =\frac{1}{n}\sum_{i=1}^{n}\nabla B_{zi}$$

(5) $$\sigma^{\prime }=\sqrt{\frac{1}{m}\sum_{i=1}^{m}\left(\sigma_{\nabla B_{zi}}-\mu \right)}$$

(6) $$\mu^{\prime }=\frac{1}{m}\sum_{i=1}^{m}\sigma_{\nabla B_{zi}}$$

(7) $$K=\mu^{\prime }+\sigma^{\prime }$$

In the formula, *σ*_∇_*_Bz_* denotes the standard deviation of the magnetic signal gradient on a single detection channel; *μ* represents the mean value of the magnetic signal gradient on a single channel; *σ*′ signifies the standard deviation of *σ*_∇Bz_ across multiple detection paths; *μ*′ indicates the mean value of the standard deviation across multiple detection paths; *K* serves as the threshold for classifying the severity of spontaneous leakage magnetic field variations; *n* denotes the number of magnetic signals between peaks and troughs of *B_z_*; and *m* represents the number of sensors.

**Figure 13:**
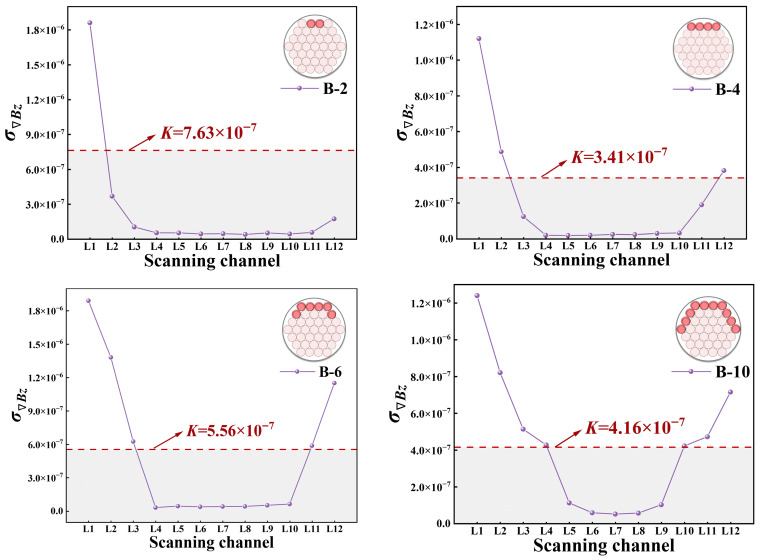
Gradient standard deviation and threshold diagram for Group B simulation conditions.

As demonstrated in Figure 13, the gradient standard deviation and threshold of the magnetic induction intensity Bz collected from the 12 scanning paths under each operating condition for Group B were calculated based on the aforementioned formula. The gradient standard deviation attained its maximum value at the corrosion centre (L1). As the circumferential width of the corrosion increased, the gradient standard deviation values at the corrosion centre along the left and right lateral directions (L2, L12) also increased accordingly. The threshold *K*, delineated by the red dashed line, was utilised to segment the 12 gradient standard deviation groups. It was determined that the regions above the threshold largely corresponded with the actual circumferential corrosion zones, providing a basis for subsequent imaging.

### 5.2. Weak Magnetic Imaging and Localisation Analysis

**Figure 14:**
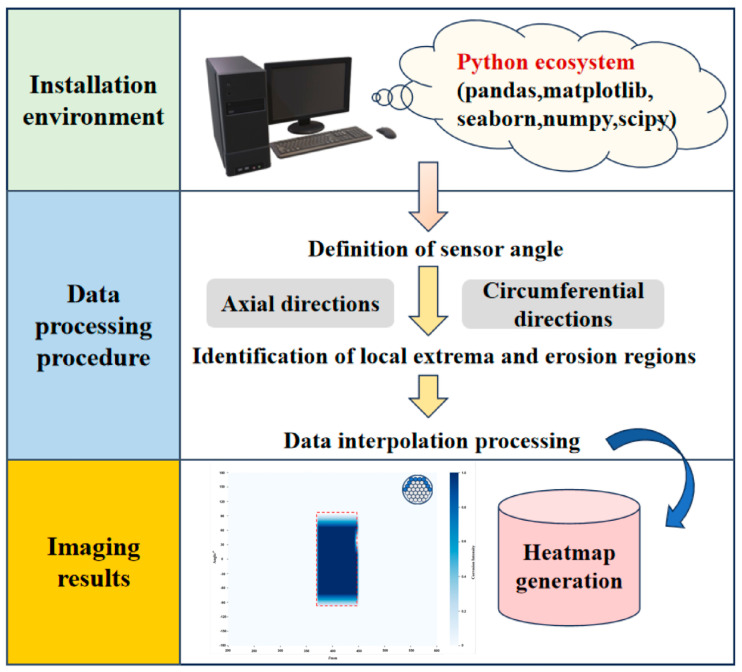
Weak magnetic imaging flowchart.

Through this study, a comprehensive weak magnetic signal imaging analysis workflow was established on the PyCharm software platform, as shown in Figure 14. This workflow encompasses the entire computational process, from the configuration of the Python environment, the definition of sensor angle, and the processing of data to the output of thermal imaging. The processing of experimental data from spontaneous magnetic leakage detection tests on parallel wire bundles under various corrosion damage states has enabled the precise visual characterisation of both axial and circumferential corrosion damage in parallel wire bundles.

**Figure 15:**
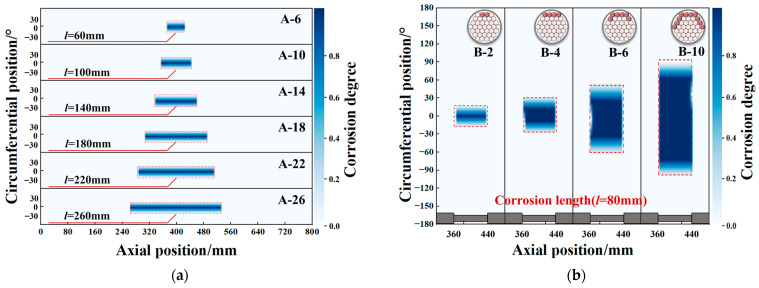
Weak magnetic signal imaging results. (**a**) Axial imaging result. (**b**) Circumferential imaging result.

In the present study, specimens from Group A were selected as representatives for the purpose of axial corrosion location weak magnetic imaging, while specimens from Group B were chosen for the purpose of circumferential corrosion location weak magnetic imaging. The specific imaging results are displayed in Figure 15. The arrangement of the 12 sensors was achieved by creating a numpy array with a 30° step size, centred at 0° with clockwise as positive. The configuration in question resulted in the sensors being arranged in a circumferential 360° configuration. By selecting the maximum and minimum values along each sensor’s scanning path, local maxima (peaks) exceeding 0.8 times the maximum value and local minima (valleys) below 0.8 times the minimum value were identified. The X-coordinates of these peak-valley pairs were then arranged in a sequential manner. In instances where the initial point corresponds to a peak (or trough) and the subsequent point is a trough (or peak), the intervening interval is designated a corrosion zone. This methodology is employed to identify axial local extrema and to mark corrosion regions. For the X-intervals corresponding to corrosion zones determined axially, the standard deviation of gradient values across all scan paths within each interval is calculated. The threshold is calculated based on the standard deviation, defined as the mean gradient standard deviation plus the standard deviation of the gradient standard deviation. The gradient standard deviation of each angular position is then compared against this threshold. Should the gradient standard deviation at any angle exceed the threshold, this would be indicative of a potential corrosion defect. Subsequently, the data from each scan path is subjected to interpolation, employing linear interpolation to generate the image.

Axial imaging (Group A) reveals a banded corrosion distribution along the axial length (increasing from A-6 to A-14), whilst circumferential imaging (Group B) shows a banded distribution along the circumferential direction (increasing width from B-2 to B-10). It is evident that both imaging modes demonstrate a high degree of correlation with predefined corrosion parameters. Pseudo-colour images generated via linear interpolation visually indicate damage severity through gradation (dark areas signifying high corrosion probability). The circumferential expansion range of B-10 is 3.2 times that of B-2, which is consistent with the circumferential expansion trend of corroded steel wires. This verifies the imaging method’s sensitivity to corrosion-induced dimensional changes. This approach provides a reliable technical means for intelligent detection of concealed damage in cable structures.

## 6. Conclusions

The present study aims to address the challenge of detecting concealed corrosion in parallel steel wire cables. In order to address this issue, a novel method of weak magnetic imaging and quantitative characterisation is proposed, based on the spontaneous magnetic leakage detection. Electrochemical corrosion tests were conducted to simulate damage conditions affecting wires between 6–26 cm axially and 2–10 wires circumferentially. A 12-channel Hall array was employed to acquire weak magnetic signals, thereby enabling systematic investigation of the mapping relationship between corrosion parameters and magnetic signal characteristics. The research systematically explored weak magnetic imaging techniques for locating corrosion within parallel steel wire cables and quantifying its severity, yielding the following principal findings:

(1) The experimental research on weak magnetic imaging of cable corrosion damage was based on sensor arrays. The purpose of employing these was to accelerate the simulation of cable corrosion. The objective was accomplished by conducting a series of electrochemical corrosion tests. The experimental findings demonstrate a robust linear correlation between axial corrosion length and the peak-to-valley distance of the normal component (R^2^ = 0.998), while circumferential corrosion width demonstrates a positive correlation with the circumferential expansion angle of the magnetic signal.
(2) The spontaneous leakage magnetic field distribution characteristics of parallel steel wire bundles were investigated systematically using finite element software to establish simulation models, with the focus being on the effects of varying circumferential corrosion widths. This finding validated the quantitative relationship between corrosion parameters and leakage magnetic field response, thereby demonstrating a positive correlation between circumferential corrosion width and the expansion range of the normal component *Bz* signal. Concurrently, the reliability of experimental data was confirmed.
(3) The present study proposes an adaptive threshold *K*, which is designed to delineate corrosion zones with high precision. This approach is predicated on the observation that the standard deviation of the magnetic signal gradient, designated hereinafter as *σ*∇*Bz*, undergoes a substantial increase in corroded areas. A weak magnetic imaging algorithm developed in Python integrates threshold segmentation with linear interpolation techniques, enabling precise localisation of corrosion regions. The imaging results evidently demonstrate the distribution characteristics of corroded areas, thereby validating the method’s feasibility for reconstructing corrosion morphology.

However, it should be noted that the present research study is subject to certain practical limitations in the context of engineering applications. In the context of inspecting large-span, high-background-noise in-service bridges, the SMFL’s resistance to interference remains a significant challenge. The statistical work involved in determining the gradient standard deviation threshold, *K*, demands high requirements for both the scanning pitch of the magnetic signal and the density of scanning channels. Linear interpolation is susceptible to the occurrence of smoothing effects at the boundaries of corrosion. Subsequent work will focus on optimising the adaptive threshold in order to reduce boundary localisation errors. Furthermore, a critical next step will be to conduct a comprehensive uncertainty and error analysis, quantifying the contributions of sensor noise, environmental fluctuations, and positioning accuracy to determine the detection limits of this system in practical applications.

## Figures and Tables

**Table 1:** Chemical composition and mechanical properties of steel wire.

Chemical Composition (%)	C	Si		Mn	S	P
	≤0.12	0.15~0.35		0.30~0.60	≤0.025	≤0.025
Mechanical Properties	Tensile Strength		Yield Strength		Elongation	
	≥1670 MPa		≥1410 MPa		≥4%	

**Table 2:** Specimen numbering and parameters.

Specimen No.	Corrosion Length *l* (cm)	Number of Corroded Wires	Corrosion Location
A-6, A-10, A-14, A-18, A-22, A-26	6, 10, 14, 18, 22, 26	4	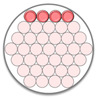
B-2	8	2	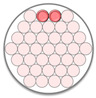
B-4	8	4	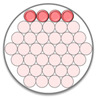
B-6	8	6	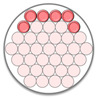
B-10	8	10	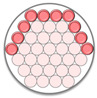

## Data Availability

The original contributions presented in this study are included in the article. Further inquiries can be directed to the corresponding author.
